# Site-directed mutagenesis of bifunctional riboflavin kinase/FMN adenylyltransferase via CRISPR/Cas9 to enhance riboflavin production

**DOI:** 10.1016/j.synbio.2024.04.011

**Published:** 2024-04-16

**Authors:** Bing Fu, Meng Chen, Xianfeng Bao, Jiajie Lu, Zhiwen Zhu, Fuyao Guan, Chuyang Yan, Peize Wang, Linglin Fu, Ping Yu

**Affiliations:** a College of Food Science and Biotechnology, Zhejiang Gongshang University, 149 Jiaogong Road, Hangzhou, Zhejiang Province, 310035, People's Republic of China; b College of Forestry Science and Technology, Lishui Vocational and Technical College, 357 Zhongshan Street North, Lishui, Zhejiang Province, 323000, People's Republic of China; c Lishui Institute for Quality Inspection and Testing, 395 Zhongshan Street, Lishui, Zhejiang Province, 323000, People's Republic of China

**Keywords:** Vitamin B_2_, FAD synthase, *Escherichia coli* BL21, Riboflavin kinase, CRISPR/Cas9

## Abstract

Vitamin B_2_ is an essential water-soluble vitamin. For most prokaryotes, a bifunctional enzyme called FAD synthase catalyzes the successive conversion of riboflavin to FMN and FAD. In this study, the plasmid pNEW-AZ containing six key genes for the riboflavin synthesis was transformed into strain R2 with the deleted FMN riboswitch, yielding strain R5. The R5 strain could produce 540.23 ± 5.40 mg/L riboflavin, which was 10.61 % higher than the R4 strain containing plasmids pET-AE and pAC-Z harboring six key genes. To further enhance the production of riboflavin, homology matching and molecular docking were performed to identify key amino acid residues of FAD synthase. Nine point mutation sites were identified. By comparing riboflavin kinase activity, mutations of T203D and N210D, which respectively decreased by 29.90 % and 89.32 % compared to wild-type FAD synthase, were selected for CRISPR/Cas9 gene editing of the genome, generating engineered strains R203 and R210. pNEW-AZ was transformed into R203, generating R6. R6 produced 657.38 ± 47.48 mg/L riboflavin, a 21.69 % increase compared to R5. This study contributes to the high production of riboflavin in recombinant *E. coli* BL21.

## 1 Introduction

Vitamin B_2_, also known as riboflavin (RF), is an essential water-soluble vitamin, and it was first isolated from milk in the late 1870s [1]. Currently, RF is widely used in the pharmaceutical, feed, cosmetic, and food industries [2]. The derivates of RF, flavin mononucleotide (FMN) [3] and flavin adenine dinucleotide (FAD) [4], are important cofactors for oxidoreductases and dehydrogenases in almost all organisms [5], and are involved in oxidative metabolism, energy metabolism, vitamin metabolism, and other processes [6,7]. RF deficiency can interfere with the maintenance of reduced glutathione, cause cellular stress, generate nonfunctional proteins, and subsequently cause many diseases [1,8,9].

In recent decades, some genetically modified strains, including *Bacillus subtilis*, *Corynebacterium ammoniagenes, Candida* spp. and *Ashbya gossypii*, were constructed for the RF biosynthesis through different strategies [2,[10], [11], [12], [13], [14], [15]]. Although wild-type *Escherichia coli* cannot accumulate RF in common conditions, it is still a promising RF-producing bacterium due to its rapid growth, clear genetic background, ease of metabolic modification, high expression of recombinant proteins and low yield of acetic acid [16]. In addition, compared to other *E. coli* strains, *E. coli* BL21 can accumulate RF under natural conditions because it has a His115Leu mutation in FAD synthase (FADS, encoded by *ribF*), causing a decrease in RibF enzyme activity and an increase in the expression of RF synthesis genes.

**Fig. 1:**
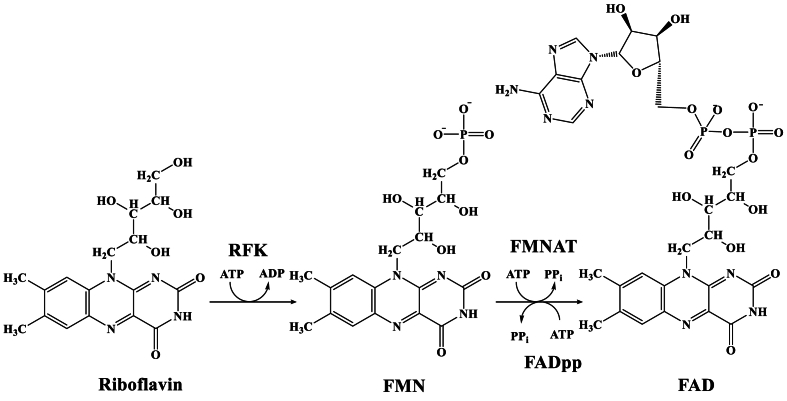
Diagrammatic representation of the successive conversion of riboflavin into FMN and FAD.

RF is first catalyzed to FMN, and then to FAD in *E. coli* BL21 by a bifunctional enzyme FADS (Fig. 1). FADS folds into two nearly independent modules, the C-terminus with riboflavin kinase (RFK) activity and the N-terminus with FMN aminotransferase (FMNAT) activity [9,17]. To increase RF production, it is a key point to reduce its conversion to FMN and FAD. Since *ribF* is an essential gene for the growth of *E. coli* [[18], [19], [20]], its knockout is lethal to the strain. Therefore, we can only reduce the enzymatic activity of FADS by some means to enhance the production of RF. A variety of metabolic engineering approaches have been applied to down-regulate FADS activity, and these operations have improved the production of RF in different microorganisms [13,21,22]. However, no information can be obtained on enhancing RF production by down-regulating FADS activity via site-directed mutagenesis in *E. coli* BL21.

In this study, a 4-isopropylbenzoicacid (cumate)-inducible plasmid pNEW-AZ containing six key genes in RF synthesis of *E. coli*, *ribA*, *ribB*, *ribC*, *ribD*, *ribE* and *zwf*, was constructed for facilitating the production of RF. Homology matching and molecular modeling were used to determine the potential mutated sites of FADS, and pETDuet-1 was employed to express mutated FADS. CRISPR/Cas9 was performed to complete the site-directed mutation of *ribF* in the *E. coli* BL21(DE3) genome to generate strain R203. The pNEW-AZ plasmid was transformed into strain R203 to obtain strain R6. Through these efforts, the titer of RF was enhanced to 657.38 ± 47.48 mg/L with 10 g/L glucose.

## 2 Materials and methods

### 2.1 Plasmids, primers, strains, media, and growth conditions

All plasmids and strains used in this study are listed in Table 1. All primers used in this study are listed in Table 2. *E. coli* DH5α was used as the host to propagate plasmid DNA, and *E. coli* BL21(DE3) was used as an expression host. The expression plasmid pETDuet-1 purchased from Novagen Co. Ltd, USA, was employed to construct mutated *ribF* and express the mutant proteins. The expression plasmid pNEW, which was purchased from FORHIGH BIOTECH, was used to express key genes of RF synthesis.

**Table 1:** Strains and plasmids.

Strains/Plasmids	Description	Source/References
**Strains**		
*E. coli* DH5a	Cloning vector, wild-Type, F^−^*φ80 lac ZΔM15 Δ(lacZYA-arg F) U169 endA1 recA1 hsdR17(rk*^*-*^*,mk*^*+*^*) supE44λ- thi -1 gyrA96 relA1 phoAe*	TaKaRa
BL21(DE3)	Expression vector, wild-Type, F^−^*ompT hsdS*_*B*_*(rB*^*-*^*mB*^*-*^*) gal dcm(DE3)*	TaKaRa
R2	E. *coli BL21* ΔsroG	Fu et al. [23]
R4	R2 containing pET-AE & pAC-Zwf	Fu et al. [23].
R5	R2 containing pNEW-AZ	this study
BF	BL21(DE3) containing pET-*ribF*	this study
B203A	BL21(DE3) containing *ribF*_*M*_-T203A	this study
B203D	BL21(DE3) containing *ribF*_*M*_-T203D	this study
B204	BL21(DE3) containing *ribF*_*M*_-I204D	this study
B210	BL21(DE3) containing *ribF*_*M*_-N210D	this study
B258A	BL21(DE3) containing *ribF*_*M*_-H258A	this study
B258D	BL21(DE3) containing *ribF*_*M*_-H258A	this study
B259	BL21(DE3) containing *ribF*_*M*_-L259D	this study
B260	BL21(DE3) containing *ribF*_*M*_-L260A	this study
B261	BL21(DE3) containing *ribF*_*M*_-D261A	this study
B299	BL21(DE3) containing *ribF*_*M*_-E299 N	this study
B303A	BL21(DE3) containing *ribF*_*M*_-R303A	this study
B303D	BL21(DE3) containing *ribF*_*M*_-R303D	this study
R203	R2 with native *ribF* replaced by mutant *ribF*^*(T203D)*^ in genome	this study
R210	R2 with native ribF replaced by mutant *ribF*^*(N210D)*^ in genome	this study
R6	R203 containing pNEW-AZ	this study
R7	R210 containing pNE-AZ	this study
***Plasmids***		
pETDuet-1	expression vector, Amp^R^, *P*_*T7-LazO*_, two MCS	Novagen
pNEW	expression vector, Kn^R^, *P*_*T5-CuO*_, P_*km*_-*cymR*	FORHIGH BIOTECH
pET-AE	pETDuet-1 with *ribC*, *ribE*, *ribB*, *ribD*, and *ribA*, *Amp*^*R*^	Fu, Ying [23]
pAC-Z	pACYCDuet-1 with *zwf, CmR*	Fu et al. [23]
pNEW-AZ	pNEW with *ribC, ribE*, *ribB*, *ribD*, *ribA,* and *zwf,* Kn^R^	this study
pET-*ribF*	pETDuet-1 containing *ribF* of BL21(DE3)	this study
*ribF*_*M*_-T203A	pETDuet-1 containing a nucleotide fragment mutated at position 203(T→A) of the *E.coli* BL21(DE3) RibF protein.	this study
*ribF*_*M*_-T203D	pETDuet-1 containing a nucleotide fragment mutated at position 203(T→D) of the *E.coli* BL21(DE3) RibF protein.	this study
*ribF*_*M*_-I204D	pETDuet-1 containing a nucleotide fragment mutated at position 204(I→D) of the *E.coli* BL21(DE3) RibF protein.	this study
*ribF*_*M*_-N210D	pETDuet-1 containing a nucleotide fragment mutated at position 210(N→D) of the *E.coli* BL21(DE3) RibF protein.	this study
*ribF*_*M*_-H258A	pETDuet-1 containing a nucleotide fragment mutated at position 258(H→A) of the *E.coli* BL21(DE3) RibF protein.	this study
*ribF*_*M*_-H258D	pETDuet-1 containing a nucleotide fragment mutated at position 258(H→D) of the *E.coli* BL21(DE3) RibF protein.	this study
*ribF*_*M*_-L259D	pETDuet-1 containing a nucleotide fragment mutated at position 259(L→D) of the *E. coli* BL21(DE3) RibF protein.	this study
*ribF*_*M*_-L260A	pETDuet-1 containing a nucleotide fragment mutated at position 260(L→A) of the *E.coli* BL21(DE3) RibF protein.	this study
*ribF*_*M*_-D261A	pETDuet-1 containing a nucleotide fragment mutated at position 261(D→A) of the *E .coli* BL21(DE3) RibF protein.	this study
*ribF*_*M*_-E299 N	pETDuet-1 containing a nucleotide fragment mutated at position 299(E→N) of the *E. coli* BL21(DE3) RibF protein.	this study
*ribF*_*M*_-R303A	pETDuet-1 containing a nucleotide fragment mutated at position 303R→A) of the *E. coli* BL21(DE3) RibF protein.	this study
*ribF*_*M*_-R303D	pETDuet-1 containing a nucleotide fragment mutated at position 303(R→D) of the *E. coli* BL21(DE3) RibF protein.	this study
pET-3a(+)-*ribC*_*opt*_	pET-3a(+) containing an optimized *ribC* of *B. subtilis*	this study
pCas	*RepA101(Ts) ori*, *Pcas–cas9*, *araBAD*,*ParaC-Red*, *lacIq*, Kan^R^	Novagen
pTargetF	sgRNA plasmid, *pij23119*, *pMB1*, Spe^R^	Novagen
sgRNA-*ribF*	*pMB1*, *aadA*, sgRNA target to the *ribF* of BL21(DE3)	this study
sgRNA-*ribCopt*	*pMB1*, *aadA*, sgRNA target to the *ribCopt* of BL21(DE3)	this study

**Table 2:** Primers used in this study.

Primers	Sequence
AE-F	GGAGATATACATGGCTAGCATGTTTACGGGGATTG
AE-R	GTTTGCGTTACCGCCATGGTATATCTCCTTCTTAATTATTTGTTCAGCAAATGGCC
Z-F	GGCCATTTGCTGAACAAATAATTAAGAAGGAGATATACCATGGCGGTAACGCAAAC
Z-R	GGCTAACGTTAACAACCGGTACCTTACTCAAACTCATTC
*ribF*-F	GGCGGATCCGATGAAGCTGATACGC
*ribF*-R	ATAGTTTAGCGGCCGCTTAAGCCGGTTTTG
T203A-F	AATTAGGGCGCGCGATAGGTTTCCCGACG
T203A-R	ATCGCGCGCCCTAATTCATCACCGTGGACTAC
T203D-F	CGCGACATAGGTTTCCCGACGGCGAATGT
T203D-R	GGAAACCTATGTCGCGCCCTAATTCATCACCGTGG
I204D-F	GAATTAGGGCGCACTGATGGTTTCCCGACGG
I204D-R	CCGTCGGGAAACCATCAGTGCGCCCTAATTC
N210D-F	GTTTCCCGACGGCGGATGTACCGCTACGC
N210D-R	GCGTAGCGGTACATCCGCCGTCGGGAAAC
H258A-F	AGTGGCGTTGTTAGATGTTGCAATGGACC
H258A-R	ATCTAACAACGCCACTTCCAGCTGCTGG
H258D-F	CTGGAAGTGGATTTGTTAGATGTTGCAATGGACCT
H258D-R	CAAATCCACTTCCAGCTGCTGGCGAATAC
L259D-F	AGTGCATGATTTAGATGTTGCAATGGACC
L259D-R	AAATCATGCACTTCCAGCTGCTGG
L260A-F	TGGCGGATGTTGCAATGGACCTTTACGG
L260A-R	TGCAACATCCGCCAAATGCACTTCCAGC
D261A-F	GTTAGCTGTTGCAATGGACCTTTACGGTC
D261A-R	TGCAACAGCTAACAAATGCACTTCCAGC
E299N–F	GATAATTTAACCGCCCGCGAATTTTTTGG
E299N–R	GGTTAAATTATCACGCGCAATCTGCGC
R303A-F	CGCCGCGGAATTTTTTGGGCTAACAAAACC
R303A-R	CAAAAAATTCCGCGGCGGTTAATTCATCACGC
R303D-F	ACCGCCGACGAATTTTTTGGGCTAACAAAAC
R303D-R	AAATTCGTCGGCGGTTAATTCATCACGCG
sgRNA-*ribF*-F	GCGCACTATAGGTTTCCCGAGTTTTAGAGCTAGAAATAGC
sgRNA-*ribF*-R	TCGGGAAACCTATAGTGCGCACTAGTATTATACCTAGGAC
Up(C)–F	TGCGTTGTGCTTACGAGCCTTTT
Up(C)-R	ATATGAATGGTTTTCATGTCTGGCTCAAAACAGTGAAAAT
Mid(C)–F	CACTGTTTTGAGCCAGACATGAAAACCATTCATATTACCC
Mid(C)-R	TGATTACATAACAGGCAGTTGCTGGATTACTATGACCCTA
Down(C)–F	TCATAGTAATCCAGCAACTGCCTGTTATGTAATCAAACCG
Down(C)-R	GTCAGGTACGGGTGCGACCAGTC
*ribC*(F)-JC-F	ACTCGGACGATTTTCACTGTTTTGAGCCAGAC
*ribC*(F)-JC-R	CGGTTCCGTGTTTCGGTTTGATTACATAACAGGC
sg- *ribC*_*opt*_-F	ACAAATTGAGTTATGTTCATGTTTTAGAGCTAGAAATAGC
sg- *ribC*_*opt*_-F	ATGAACATAACTCAATTTGTACTAGTATTATACCTAGGAC
Up(F)–F	GACGGTCCACGATCGGTTGCATTTC
Up(F)-R	CCGCGTATCAGCTTCATGTCTGGCTCAAAAC
Mid(F)–F	GTTTTGAGCCAGACATGAAGCTGATACGCGG
Mid(F)-R	TCGGTTTGATTACATAACAGGCTTAAGCCGGTTTTGTTAG
Down(F)–F	CTAACAAAACCGGCTTAAGCCTGTTATGTAATCAAACCGA
Down(F)-R	GGCTTCAGTTTTGAAGTCCATGGTCAGGTAC

Note: Underlined letters indicate the restriction endonuclease-digested sites.

Luria–Bertani (LB) medium (tryptone 10 g/L, yeast extract 5 g/L, and NaCl 10 g/L) was used for strain propagation and screening of RF-producing strains. Appropriate antibiotics were added to LB when necessary. Unless specifically mentioned, all the strains were incubated in LB at 37 °C under good aeration.

The FastPure plasmid mini kit, FastPure gel DNA extraction mini kit, FastPure® bacteria DNA isolation mini kit, and ClonExpress II one-step cloning kit were purchased from Vazyme Biotech (Nanjing, China). PrimeSTAR® HS (Premix), the competent cell preparation kit and restriction endonucleases (*Bam*H I, *Not* I, *Kpn* I, *Nhe* I and *Dpn* I) were purchased from Takara Bio (Dalian, China). Ni-NTA His Bind Resin was purchased from Sangon Biotech (Shanghai, China). The BCA protein assay kit, SDS‒PAGE preparation kit, and standard samples (RF & FMN & FAD) were purchased from Shanghai Biotech (Shanghai, China). Primer synthesis and sequencing were performed to Shanghai Biotech, China.

### 2.2 Construction of expression plasmids

The plasmids pET-AE and pAC-Z harboring the nucleotide fragments *ribC*-*ribE*-*ribB*-*ribD*-*ribA* and *zwf* for the synthesis of RF were constructed before in our laboratory [23]. To construct an expression plasmid with cumate as an inducer, primers AE-F/R were used to amplify pET-AE to obtain the gene fragment *ribC*-*ribE*-*ribB*-*ribD*-*ribA*. Meanwhile, Z-F/R was used to amplify the plasmid pAC-Z to obtain the nucleotide fragment *zwf*. These two fragments were ligated by overlap PCR with the primers AE-F/Z-R, and the products were extracted and homologously recombined with a linear fragment of pNEW that had been previously double digested by *Kpn* I and *Nhe* I (Supplementary Fig. S1 a).

To obtain the plasmid to express RibF, the BL21 (DE3) genome DNA was extracted using the FastPure® bacteria DNA isolation mini kit and used as a PCR template and amplified with *ribF*-F/R as the primers. The PCR products were purified by a FastPure Gel DNA extraction mini kit, and homologous recombination was performed to insert the *ribF* gene fragment into pETDuet-1 (digested by *Bam*H I & *Not* I) by a ClonExpress II one-step cloning kit, named pET-*ribF* (Supplementary Fig. S1 b). Reserve PCR was performed to obtain mutated *ribF* with pET-*ribF* as a template, and the PCR products were transformed into *E. coli* DH5α competent cells after digestion by *Dpn* I. Positive colonies were screened out by sequencing and stocked in glycerol tubes at −80 °C. The constructed plasmids were respectively transformed into *E. coli* cells by heat shock except for CRISPR gene editing.

### 2.3 Preparation of cytosolic fractions and SDS–PAGE analysis of the target proteins

The expression plasmids were transformed into *E. coli* BL21(DE3) competent cells to express wild-type or mutated RibF. Recombinant *E. coli* BL21 (DE3) was incubated in 50 mL LB medium containing 100 μg/mL ampicillin at 37 °C. When the OD_600_ value reached 0.6–0.8, isopropyl β-d-thiogalactopyranoside (IPTG) with a final concentration of 100 mM was added into LB medium to induce the expression of target proteins at 28 °C overnight. The cells were collected by centrifugation at 13,400×*g* for 10 min, and after removing the supernatant, the obtained cells were resuspended in 1 × PBS buffer (pH 7.4). Proteins were released by ultrasonic treatment (SCIENTZ-II D, China): work 2 s, pause 5 s, 150 W, 10 min. The resulting crushing solution was centrifuged (13,400×*g*, 10 min, 4 °C), and the pellet was further resuspended in 1 × PBS buffer.

To extract the crude proteins from mutated strains, single colonies of mutated strains were incubated with 5 mL LB medium overnight at 220 rpm and 37 °C. The cultures were added to 100 mL LB medium. When the OD_600_ value reached ∼1.0, the cells were collected by centrifugation. The crushing procedure was described above. The supernatants were stored in a −80 °C refrigerator for the subsequent analysis.

SDS‒PAGE (12 % separating gel and 5 % stacking gel) was performed to identify the expression of target proteins. Coomassie brilliant blue G-250 was used to visualize the resolved proteins.

### 2.4 Purification of target proteins

For purification of the target proteins, Ni^2+^-NTA column chromatography was performed. The supernatants prepared from each cell culture were applied to a Ni^2+^-Sepharose column equilibrated with binding/washing buffer (20 mM Tris-HCl, 500 mM NaCl, 1 mM PMSF, and 30 mM imidazole, pH 7.4) at a flow rate of 1 mL/min. Subsequently, the column was washed with the same buffer to remove other proteins, and the histidine-tagged proteins were eluted with elution buffer (20 mM Tris-HCl, 500 mM NaCl, and 300 mM imidazole, pH 7.4).

The concentrations of the purified proteins were determined using the BCA kit, following the manufacturer's procedures.

### 2.5 Detection of quaternary organizations *in vitro*

There is no evidence as to whether FADS from *E. coli* (*Ec*FADS) also forms a dimer-trimer conformation [17]. To determine the potential assembly of *Ec*FADS in solution, a protein crosslinking reaction was performed. Samples containing 30 ng of *Ec*FADS (42 μM, 20 μL) in 20 mM PIPES, pH 7.0, were incubated for 30 min at 25 °C in both the absence and presence of 3.5 μM bis(sulfosuccinimidyl)suberate (BS_3_) crosslinker (Aladdin, China). The crosslinking reaction was stopped by the addition of 0.5 M Tris/HCl, pH 8.0, until a final concentration of 50 mM [24]. Reaction products were then resolved by SDS–PAGE (15 % separating gel and 5 % stacking gel).

### 2.6 Sequence alignment and docking simulations

ClustalW was used to align four FADS amino acid sequences derived from *C. ammoniagenes*, *Mycobacterium tuberculosis*, *E. coli* BL21(DE3), and *B. subtilis*. The results of the comparison were downloaded, and then make a graph using ESPript 3.0. For docking simulations, the structure model of *Ec*FADS was built via swiss-model [https://swissmodel.expasy.org/] by using the D298E mutant of FAD synthetase from *C. ammoniagenes* (SMTL ID: 5fo0.1 [https://swissmodel.expasy.org/templates/5fo0]) as a template. The Autodock Vina software was used to simulate the binding of the *Ec*FADS model to the substrate RF. An AMBER force-field based algorithm was used to accurately forecast the free energy of binding. The ligands final conformations were chosen based on the estimated binding energies. The following docking box settings were configured when using the local docking mode: The coordinates of the center were as follows: center x = −3.785, center_y = −32.715, and center_z = −25.368. The dimensions of the object are: size_x = 62, size_y = 60, and size_z = 56, with grid spacing of 0.375 Å, and forecasts of 20 poses. The Ligplot software was used to view the docking results. The active central sites and conserved amino acid sites were identified based on the results of molecular docking and homology matching.

### 2.7 Measurement of the activity of RFK

As RF is successively converted to FMN and FAD via *ribF*-encoded RFK and FMNAT, reducing RFK activity decreases the consumption of RF. Thus, RFK activity was measured to compare the effect of different mutants.

Before detecting the activity of RFK, the concentration of purified proteins was measured by the BCA kit and using bovine serum albumin as a standard. RFK activity was measured in a final volume of 1 mL of potassium phosphate buffer (pH 7.5, containing 50 μM RF, 3 mM ATP, 15 mM MgCl_2_, and 10 mM Na_2_SO_3_). The mixture was preincubated at 37 °C for 5 min, and 20–60 nM enzymes (higher concentrations used for variants with very low activities) [4] were added into the mixture to initiate the reaction. The mixture was then incubated at 37 °C for 10 min and then heated at 100 °C for 5 min to stop the reaction [4,25]. A total of 63 μg crude enzymes were added to the mixture as described above when measuring the RFK activity of the mutant strains, and the reaction time was prolonged to 30 min. Centrifugation was performed to remove the proteins, and the FMN concentration in the supernatant was analyzed by HPLC.

To further determine the steady-state rates of WT *Ec*FADS and mutants, the RFK activity was measured at increasing concentrations of RF (1–50 μM) and a saturated concentration of ATP, and at increasing concentrations of ATP (0.01–0.8 mM) and a saturated concentration of RF [26]. The data obtained were fitted to the Michaelis-Menten equation to obtain Michaelis-Menten constant (*Km*) and catalytic rate (*kcat*) [26].

### 2.8 CRISPR/Cas9 genomic editing

CRISPR/Cas9 was carried out to achieve the replacement of native *ribF* with mutated *ribF* in the *E. coli* BL21(DE3) genome. *RibF* is an essential gene for *E. coli* [20] and avoids cleavage of the repair template by pCas due to the small difference between the inserted gene and the native gene. Two-step genomic editing was performed. First, the *ribF* gene on the *E. coli* BL21(DE3) genome was replaced by a fragment consisting of *ribC* from *B. subtilis* (encoding a flavonase/FAD-synthase) and a 63 bp sequence immediately (GTAGTAAAGGCGCTTCAATCATGAACATA ACTCAATT TGTAGGGTCATAGTAATCCAGCAACT) followed by *ribC* termination codon TAA. The fragment was synthesized by Sangon Biotech and named *ribC*_*opt*_. This fragment was inserted into pET-3a (+), generating pET-3a(+)-*ribC*_*opt*_. Second, *ribC*_*opt*_ was replaced by the appropriate mutated *ribF* to generate engineered strains.

The procedure of CRISPR/Cas9 was described in detail [27]. sgRNA-*ribF* was obtained by reverse PCR with sgRNA-*ribF*-F/R as primers and pTargetF as the template. PCR products were treated with *Dpn* I and then transformed into *E. coli* DH5α competent cells. After heat shock and incubation, the culture was spread on LB plates with 50 μg/L spectinomycin for incubation at 30 °C overnight. The single colony was selected and cultured in 5 mL LB with 50 μg/L spectinomycin. The plasmids were extracted and sequenced by Shanghai Biotech. The primers Up(C)–F/R and Down(C)–F/R were used to amplify the upstream and downstream homology arms [Up(C) & Down(C), ∼500 bp] with the *E. coli* BL21(DE3) genome DNA as the template. The primers Mid(C)–F/R were used to obtain the nucleotide fragment *ribC*_*opt*_ with the plasmid pET-3a(+)-*ribC*_*opt*_ as the template. Overlap PCR was performed to acquire the donor DNA for repair, and the nucleotide fragments Up(C), Down(C), and *ribC*_*opt*_ were used as templates, with Up(C)–F/Down(C)-R as primers.

pCas was transformed into *E. coli* BL21(DE3)-Δ*sroG* (R2) [23] to generate R2*-*pCas. Electroporation-competent R2*-*pCas cells were produced as described previously [23]. Then, 400 ng sgRNA-*ribF* and 2.0 μg donor DNA were added to 100 μL electroporation-competent cells, and after 30 min of incubation on ice, electroporation was performed. After 1.5 h incubation with 900 μL LB medium without antibiotic at 30 °C and 140 rpm, the culture was spread on LB plates with 50 μg/L spectinomycin and 50 μg/L kanamycin and incubated overnight at 30 °C. Positive clones were screened by colony PCR and further verified by sequencing.

After curing the sgRNA-*ribF* with 0.5 mM IPTG, the positive colony containing pCas was subjected to the next operation. sgRNA-*ribC*_*opt*_ which targets the 63 bp sequence was obtained by reverse PCR, with pTargetF as the template and sg-*ribC*_*opt*_-F/R as primers. The primers Up(F)–F/R and Down(F)–F/R were used to amplify *E. coli* BL21 (DE3) genomic DNA, and ∼500 bp upstream and downstream homologation arms were obtained, named Up(F) and Down(F). The intermediate nucleic acid fragment was obtained by amplifying a suitable mutant *ribF*-containing plasmid with primers Mid(F)–F/R. The three fragments were amplified with primers Up(F)–F/Down(F)-R to obtain donor DNA. The subsequent electroporation process was carried out as described previously. Subsequently, sgRNA-*ribC*_*opt*_ and pCas were sequentially cured by IPTG induction and 42 °C incubation, respectively.

Gene transfection instrument (Scient-2C, SCIENTZ, China) was applied to transform sgRNA and donor into electroporation-competent cells. The experimental parameters were as follows: voltage, 1500 V; capacitance, 25 μF; resistance, 400 Ω; gene pulser cuvette, 2 mm.

### 2.9 Transformation of the expression plasmids

Single colonies of *E. coli* BL21(DE3) gene-edited bacteria were picked and incubated in antibiotic-free LB medium to an OD_600_ value between 0.35 and 0.6. Competent cells of *E. coli* BL21(DE3) gene editing bacteria were prepared on ice using the competent cell preparation kit (Takara). The expression plasmids were transformed into competent cells by heat shock, and a single colony was used for RF production.

### 2.10 Detection methods

OD_600_ was detected by a PGENERAL new century T6 spectrophotometer (Beijing, China). The 3,5-dinitrosalicylic acid method was used to measure the concentration of residual glucose [23]. The concentration of FMN was measured by HPLC (Agilent 1260), and detection conditions were as follows: mobile phase, methanol–100 mM formic acid–100 mM ammonium formate (25: 75, vol/vol, pH 3.7); flow rate, 1.2 mL/min; column, ZORBAX SB-C18 4.6 × 250 mm, 5 μm; injection volume, 10 μL. Detection was carried out with a fluorescence detector (1260 Infinity II fluorescence detector; excitation, 470 nm; emission, 530 nm). RFK activity is expressed as millimoles per gram of protein catalyzing the production of FMN from RF per unit time [28]. The RF concentration was detected as described previously [23]. The concentration of acetic acid in the fermentation product was determined by HPLC (Agilent 1260), and the detection conditions were as follows: mobile phase, methanol-0.1 % sulfuric acid (15: 85, vol/vol); flow rate, 1.0 mL/min; column, Agilent Eclipse plus C18 4.6 × 250 mm, 5 μm; injection volume, 10 μL; temperature, 30 °C; and detection by ultraviolet detector at 205 nm [29].

### 2.11 Statistical analysis

All experiments were performed in triplicate, and the results are presented as the means ± SDs. Statistical analysis was performed by SPSS 17.0 software. Data were graphed using Origin 8.5 software. A plasmid construction schematic map was drawn with ChemDraw Professional 2017 software.

## 3 Results

### 3.1 Optimization of the concentration of the inducer cumate

It is generally accepted that the pET series vectors can only be expressed in *E. coli* BL21(DE3) hosts containing T7 RNA polymerase. However, in this study, when the expression plasmid pET-AE was transformed into *E. coli* DH5α, a certain amount of RF was detected in the fermentation broth of recombinant strain even in the absence of the inducer IPTG (∼20 mg/L RF), indicating that the specificity of the T7 promoter is not ideal. Furthermore, the leaky expression of the pET series vectors severely compromised the expression of target genes [30], as the presence of flavin produced severe oxidative stress [31]. For these reasons, we chose to replace the pET series vector with the pNEW vector, which has the same expression intensity as the pET vector series, is more tightly regulated and has a cheaper induction agent (cumate) [32].

After successful construction of pNEW-AZ, the plasmid was transformed into R2 to generate strain R5. To investigate the effect of RF production at different concentrations of cumate, the engineered strain R5 was incubated in 5 mL LB medium (with 50 mg/L kanamycin). After overnight incubation, 2 % (v/v) culture was added to 50 mL LB medium in a 250 mL shake flask. When the OD_600_ value reached ∼0.6–0.8, 1, 5, 10, 25, 50, 100, 200, and 500 μM cumate was added to 50 mL LB medium to induce the expression of target genes.

**Fig. 2:**
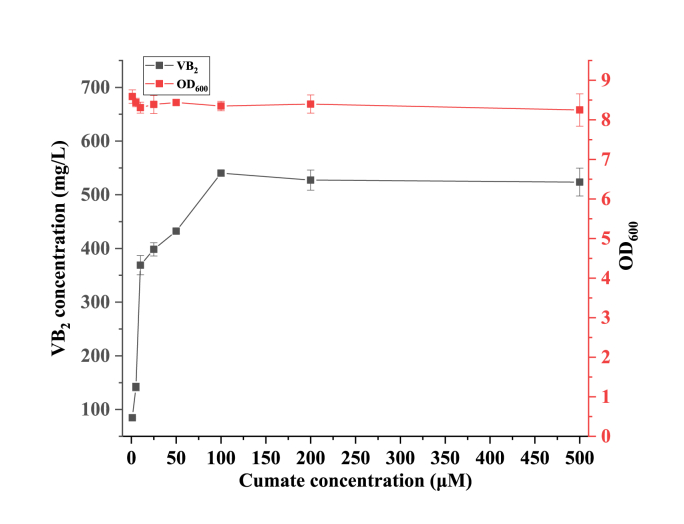
Riboflavin production and OD_600_ of the R5 strain at different cumate concentrations.

As shown in Fig. 2, after 72 h of incubation, the highest RF titer was 540.23 ± 5.40 mg/L with an induction concentration of 100 μM (with a yield of 59.42 ± 0.59 mg RF/g glucose), which was 10.61 % higher than that of strain R4 (produced 488.40 ± 11.18 mg/L RF in LB medium) [23]. The increase in RF production may not only be due to reduced leakage expression, but also to a reduction in the number of plasmids and thus a lower metabolic burden. When cumate concentrations were between 1 and 10 μM, RF titers showed a linear increase with little difference in OD_600_ values. When cumate concentrations were between 10 and 100 μM, the RF titers increased further with increasing inducer concentrations. The high concentrations of inducer (≥500 μM) were not effective in raising the RF titer. Because cumate is an acidic compound, too high concentration will lower the pH of the medium, which in turn will affect the growth of the strain.

### 3.2 Protein cross-linking experiments and determination of RibF mutation sites

**Fig. 3:**
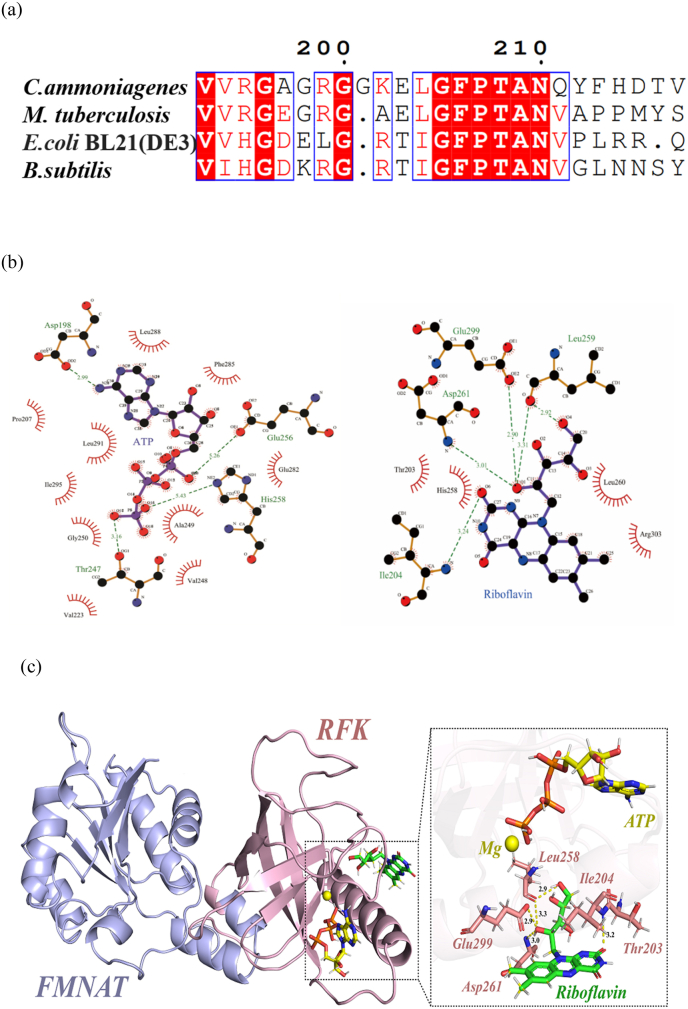
Identification of key amino acid sites for RFK module of WT *Ec*FADS. a. Result of RibF amino acid sequence alignment from four different microorganisms. b. A two-dimensional diagram of the molecular docking results of substrates (left: ATP; right: riboflavin) interaction with the RFK module of WT *Ec*FADS. The hydrogen bonds between the amino acid and the substrate are represented with dotted lines (green). The hydrophobic bonds are represented with an eyelash shape. c. A three-dimensional diagram of the molecular docking results of substrates interaction with the RFK module of WT *Ec*FADS. RF and ATP ligands are shown as sticks and colored with carbons in green and yellow, respectively. The Mg^2+^ cation is shown as a yellow sphere. The FMNAT module is blue and the RFK module is pink.

FADS amino acid sequences derived from *C. ammoniagenes*, *M. tuberculosis*, *E. coli* BL21(DE3), and *B. subtilis* were aligned, and a conserved sequence GFPTAN (205–210, *E. coli* numbering) was observed (Fig. 3a), in which PTAN (207–210) is proposed to stabilize the metal ion and the phosphate groups of the ATP: Mg^2+^ substrate during RFK catalysis [4].

**Fig. 4:**
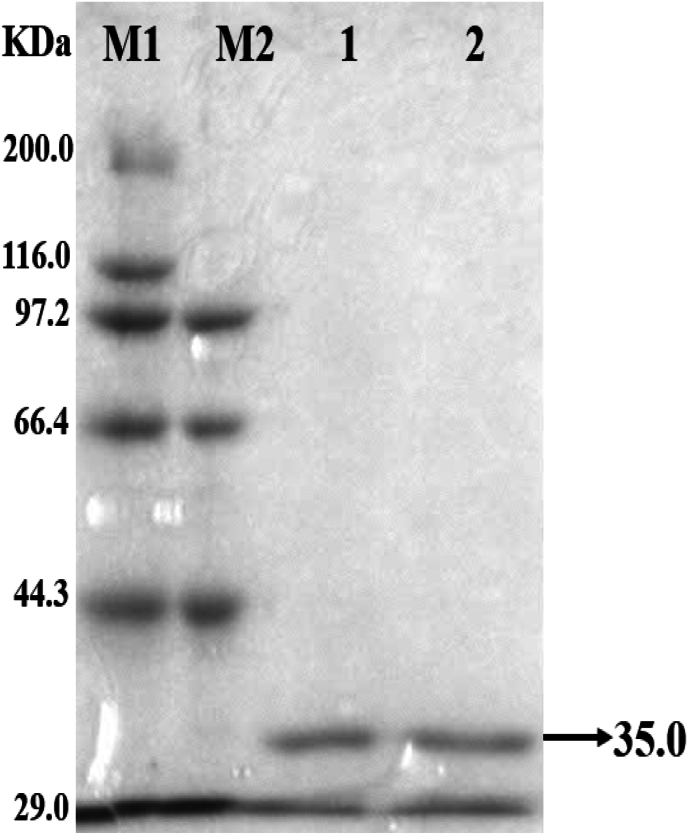
*Ec*FADS *in vitro* protein cross-linking assay. Lane M1: protein molecular weight marker (broad); Lane M2: protein molecular weight marker (low); Lane 1: protein cross-linking products without BS_3_; Lane 2: protein cross-linking products containing BS_3_.

FADS from *C. ammoniagenes* (*Ca*FADS) mainly stabilizes in hexameric assemblies in solution in a dimer-of-trimers conformation [9]. In this study, *Ec*FADS was found to be presented in solution as a monomer (trace amounts of multimeric forms may be present) by protein cross-linking experiments *in vitro* (Fig. 4). Although there are differences in the way these two FADS are assembled in solution, the system still recommends *Ca*FADS as a template for molecular docking, and the binding energy of *Ec*FADS co-docked to the substrates ATP and RF was −9.825 kcal/mol. A total of fourteen amino acids were identified in the active center bound to ATP, of which four amino acids (D198, T247, E256, and H258) formed hydrogen bonding interactions and ten amino acids (P207, V223, V248, A249, G250, E282, F285, L288, L291, and I295) formed hydrophobic interactions (Fig. 3b and c). The molecular docking results of substrate RF showed that the following eight amino acids were present around the active site, in which four amino acids form hydrophobic interactions (T203, H258, L260, and R303), and the other four form hydrogen bonding interactions (I204, L259, D261, and E299) (Fig. 3b and c). Due to time constraints, this study tried to reduce RFK enzyme activity by mutating key amino acids that interact with the substrate RF. Ultimately, the mutant sites T203A, T203D, I204D, N210D, H258A, H258D, L259D, L260A, D261A, E299 N, R303A, and R303D were determined.

To obtain RibF variants, pET-*ribF* was constructed first, *ribF*-F/R was used to amplify *ribF* with the *E. coli* BL21(DE3) genome DNA as the template, and the gel recovery product was homologously recombined with a linear fragment of pETDuet-1 that had been previously digested and recovered. Mutant plasmids *ribF*_M_-T203A, *ribF*_M_-T203D, *ribF*_M_-I204D, *ribF*_M_-N210D, *ribF*_M_-H258A, *ribF*_M_-H258D, *ribF*_M_-L259D, *ribF*_M_-L260A, *ribF*_M_-D261A, *ribF*_M_-E299 N, *ribF*_M_-R303A, and *ribF*_M_-R303D were acquired by reverse PCR using corresponding primers. After digestion by *Dpn* I, all products were transformed into *E. coli* BL21(DE3) competent cells, and the single colonies were respectively named B203A, B203D, B204, B210, B258A, B258D, B259, B260, B261, B299, B303A and B303D*. E. coli* BL21(DE3) containing pET-*ribF* was named BF.

### 3.3 Expression and purification of the RibF variants and detection of RFK activity

The above strains were used to induce the expression of the target proteins by IPTG, and the supernatants after cell crushing were analyzed by SDS‒PAGE. According to SDS‒ PAGE analysis (Supplementary Fig. S2), target proteins with a molecular weight of 35 kDa were obtained in all strains, which was consistent with the expected value, indicating that proteins were correctly expressed in all thirteen strains. Target proteins were successfully purified from all strains by Ni^2+^-NTA columns, except for strain B259 (Supplementary Fig. S3). The mutation at L259D may have caused a dramatic change in spatial structure, allowing 6 × His to be encapsulated. Therefore, eleven different purified mutant RibF proteins were used for comparison of RFK activity.

Detection of RFK activity was carried out with a fluorescence detector. The RFK activity of different mutated proteins is shown in Table 3. The single amino acid mutations that caused complete loss of enzyme activity were I204D, H258A/D, and R303D. Compared to WT RibF, the RFK enzyme activities of the three RibF mutated proteins, T203D, N210D and R303A, were decreased by 29.90 %, 89.32 % and 15.92 %, respectively. The other three mutated proteins (L260A, D261A and E299 N) showed negligible changes in the RFK enzyme activity, with decreases by 2.14 %, 1.94 %, and 1.55 %, respectively. Surprisingly, the RFK enzyme activity of the T203A mutant protein was increased by 103.11 %.

**Table 3:** Comparison of RFK activity of different mutant proteins.

Mutant Protein number	RFK enzyme activity [mM/(min⋅g protein)]	Increase/decrease in RFK enzyme activity compared to WT(%)
WT	5.15 ± 0.12^b^	/
T203A	10.46 ± 1.31^a^	+103.11
T203D	3.61 ± 0.04^c^	−29.90
I204D	ND	/
N210D	0.55 ± 0.05^d^	−89.32
H258A	ND	/
H258D	ND	/
L260A	5.04 ± 0.04^b^	−2.14
D261A	5.05 ± 0.05^b^	−1.94
E299 N	5.07 ± 0.05^bc^	−1.55
R303A	4.33 ± 0.21^c^	−15.92
R303D	ND	/

Note: ND indicates that no enzyme activity was detected. Letters indicate significant differences (*P* < 0.05).

As *ribF* is an essential gene, the mutation site with the greatest reduction in RFK activity cannot simply be selected. The RFK steady-state parameters suggest that the T203D and N210D may be used for the subsequent genomic mutations (Table 4). Therefore, we chose the two mutation sites to complete mutations on the R2 genome to study the effects of different mutations on RF production and strain growth.

**Table 4:** Steady-state kinetic parameters for the RFK activity of WT, T203D, and N210D.

Mutant Protein number	*k*cat (min^−1^)	*K*RF m (μM)	*k*cat (min^−1^)	*K*ATP m (μM)
WT	49.93 ± 8.00	20.17 ± 4.23	41.70 ± 4.16	13.81 ± 4.96
T203D	39.85 ± 18.87	23.63 ± 8.88	28.57 ± 2.15	10.19 ± 2.55
N210D	5.05 ± 1.20	55.33 ± 26.57	7.56 ± 1.21	91.12 ± 36.67

### 3.4 Replacement of native ribF with mutant ribF through CRISPR/Cas9

The positive colonies were screened by colony PCR with *ribC*(F)-JC-F/R as primers, and positive clones yielded a 1080 bp band, while negative clones yielded a 1008 bp band (Supplementary Fig. S4). The positive colonies were further sequenced by Sangon Biotech (Shanghai, China).

The primers Mid(F)–F/R were used to amplify *ribF*_*M*_-T203D and *ribF*_*M*_-N210D to obtain the nucleotide fragments Mid(F)203 and Mid(F)210. DNA repair templates were obtained by using primers Up(F), Down(F), and *ribF*_*M*_-T203D/*ribF*_*M*_-N210D as templates. After CRISPR/Cas9 genomic editing, the positive colonies were screened by sequencing, and designated as R203 and R210, respectively.

### 3.5 Effect of genomic ribF mutations on RFK activity and RF production

The RFK activity of different mutated strains is shown in Table 5. The most dramatic decrease in RFK activity was observed in strain R210, with a 78.48 % reduction. The same mutation on *Ca*FADS was also observed with an 89.57 % reduction [4]. Compared to other strains (R2 and R203), the growth rate of R210 was severely impaired, and it was easy to understand that RFK reduction affected normal physiological functions because FMN and FAD were key cofactors.

**Table 5:** Comparison of RFK activities of different mutated strains.

Strain number	RFK enzyme activity [μM/(min⋅g protein)]	Increase/decrease in RFK enzyme activity compared to R2 (%)
R2	16.73 ± 0.45^a^	/
R203	12.67 ± 0.76^b^	−24.27
R210	3.60 ± 0.53^c^	−78.48

Note: Letters indicate significant differences (*P* < 0.05).

To determine the ability of the mutated strains to produce RF, pNEW-AZ was transformed into strains R203 and R210 to generate engineered strains R6 and R7, respectively. As with strain R210, the growth rate of strain R7 was also inhibited. FMN and FAD are hydrophilic and do not pass the plasma membrane, and both of these RF derivates might be hydrolyzed to form RF and then transform into cells from LB medium [33]. To restore the growth of the engineered strain R7 and facilitate the production of RF, RF at 20 mg/L was added to the LB medium to compensate for the lack of FMN/FAD before cumate induction. After 72 h of fermentation in LB medium with 10 g/L glucose (containing 50 μg/L kanamycin), the RF titers of different engineered strains were determined, and are shown in Table 6. R6 produced 657.38 ± 47.48 mg/L RF, with a yield of 72.30 ± 5.21 mg RF/g glucose, and with a 21.69 % increase compared to R5. R7 produced only 94.27 ± 0.75 mg/L RF. Meanwhile, although RF addition enhanced strain growth to some extent, the growth rate was still slower than that of the other strains. Flavoproteins with FMN/FAD as coenzymes are involved in the dehydrogenation of a variety of metabolites, in one- and two-electron transfer from and to redox centers, and in the activation of oxygen for oxidation and hydroxylation reactions [34]. The N210D mutation severely decreased RFK activity, and the lack of FMN/FAD largely affected the physiological functions of the engineered strain and thus the synthesis of RF, even when supplemented with RF in medium.

**Table 6:** Comparison of fermentation parameters between R6 and R7 strains.

Strains	OD_600_	RF titer (mg/L)	Acetic acid concentration(g/L)
R6	7.23 ± 0.24^a^	657.38 ± 47.48^a^	2.38 ± 0.18^a^
R7	1.33 ± 0.02^b^	94.27 ± 0.75^b^	0.53 ± 0.12^b^

Note: Letters indicate significant differences (*P* < 0.05).

## 4 Discussion

In recent years, because RF production by microbial fermentation is more economical and environmentally friendly than chemical and semi-chemical synthesis, several RF production strains, which are mainly based on the use of *B. subtilis*, *A. gossypii*, *Candida famata*, and *E. coli*, have been developed and applied on an industrial scale to obtain RF [35,36]. As a model organism, *E. coli* has the advantages of a clear metabolic background, rapid growth, low maintenance metabolism, and mature molecular tools suitable for its genetic manipulation, especially the ability of *E. coli* BL21 (DE3) to accumulate RF.

The T7 expression system is widely used to express target proteins and obtain target products. However, its expensive inducer IPTG limits its application in large-scale fermentation [37]. In addition, it suffers from severe leaky expression that may affect the yield of the target product [38,39]. The T7 terminator that comes with the pET series of plasmids has only 74 % termination efficiency, resulting in read-through [40]. This read-through can affect the expression of downstream elements such as antibiotic resistance genes, plasmid copy number control elements, or repressor proteins used to lower basal expression levels [41]. Cumate is nontoxic to the host, inexpensive, and a carbon source-independent inducer, which provides an economical option for the large-scale production of valuable proteins and chemicals [37]. To reduce leakage expression as well as realize large-scale production of RF at a later stage, we inserted six key genes of RF synthesis into the pNEW vector. The R5 strain transformed with pNEW-AZ showed a 10.61 % increase in RF production compared to the previous R4 strain based on the pET system for RF production. This result suggests that the cumate-induced expression system can be effectively applied in the production of target products, including RF. In the present study, it was found that although cumate is nontoxic, too high concentration apparently lowers the pH of the medium, which in turn has some negative effects on the fermentation, especially on the growth rate of the organisms.

The main function of prokaryotic FADS is to provide flavin cofactors and, at the same time, to maintain intracellular flavin and flavoprotein homeostasis [42]. Different sources of FADS differ in their enzymatic properties, particularly *Ca*FADS, and the differences are mainly in the catalytic efficiency of the enzyme (including RFK, FMNAT, or FADpp), whether it produces substrate or product inhibition, the redox environment required to produce enzymatic activity, the arrangement of the active center, and the way the protein polymerizes in solution [24,26]. These differences allow FADS to effectively regulate intracellular flavin homeostasis in different cells.

Both I204 and H258 were involved in the formation of the important protein-ligand interactions, leading to the formation of the hydrogen bond and Pi electron clouds. In *Ec*FADS, H258 was not only connected to the substrate ATP through hydrogen bonding at the active center, but also participated in the formation of the substrate RF active center through hydrophobic interaction. Thus, mutations of H258A and H258D resulted in a complete loss of RFK enzyme activity. The mutation in R303 affects the α-helix structure, resulting in large changes in the protein conformation and consequent reduction or loss of the RFK enzyme activity. The N210D mutation can disrupt the β-fold motif in the main structure of the protein, resulting in a change in the conformation of protein which makes the RFK activity decreased dramatically. The T203A mutation greatly enhances the RFK enzyme activity. Since alanine is more hydrophobic than threonine, the mutant in this locus promotes the formation of hydrophobic forces, which in turn enhances the RFK enzyme activity. L260, D261, and E299 are located in random coiling. Therefore, the associated mutations have little effect on the overall protein structure as well as the RFK activity.

Asn and Thr residues play an important role in coordinating cations at the active sites of kinases [3]. In the study of RFK activity of the mutated proteins, the mutation of N210D greatly reduced RFK activity, which not only affects the growth of the strain, but also further affects RF production. The mutation of T203A enhanced the RFK activity by 103.11 %, whereas the mutation of T203D decreased the RFK activity by 29.30 %. In *Ca*FADS, T208 and N210 provide the RFK active-site geometry for binding and catalysis, and mutations at these two sites substantially reduce the RFK activity and modulate the binding parameters at the FMNAT active site of *Ca*FADS, altering the catalytic efficiency in the transformation of FMN into FAD [4]. FMNAT activity was not measured in our study, and it is not known whether mutations in amino acids in the C-terminal RFK module of *Ec*FADS will affect the FMNAT module. Lin et al. [21] reduced the *ribF* expression level by 35.17 % and elevated RF production by 77.05 % by replacing native RBS. Hu et al. [13] knocked down the expression of the *ribF* by synthetic regulatory small RNA, which enhanced RF production by up to 132.02 % compared to the basic strain WY0S. In contrast to the results of this study, mutation of genomic T203D reduced RFK enzyme activity by 24.27 %, while RF production was elevated by 21.69 %, and the RF titer reached 657.38 ± 47.48 mg/L. The first two operations regulate the entire FADS bifunctional enzyme activity, while the mutation in the C-RFK module in this study regulates mainly RFK activity. This may explain why the first two operations are more likely to increase RF production. Xu et al. [16] constructed an engineered strain RF18S that could produce 387.6 mg/L RF with 10 g/L glucose. Another strain LS02T was constructed by Liu et al. [14] which produced 667 mg/L of RF when cultivated in MSY medium supplied with 10 g/L glucose. The strain *E. coli* RF05S could produce 585.2 ± 13.6 mg/L RF [21]. Compared to these strains, our constructed strain in this study produced 657.38 ± 47.48 mg/L RF. Thus, our study might provide a basis for the future industrial production of RF.

## 5 Conclusion

In summary, the vector pNEW-AZ constructed on the basis of the pNEW plasmid was able to enhance RF yield and reduce the inducer cost. We identified nine possible key amino acid residues in the RFK activity center of *Ec*FADS by homology matching and molecular docking, selected two amino acid sites by RFK activity assay, and obtained two genetically engineered bacteria by subsequent CRISPR gene editing based on the modified R2 strain. After transformation of the plasmid pNEW-AZ, fermentation in LB medium containing 10 g/L glucose yielded 657.38 ± 47.48 mg/L RF, which was elevated by 34.60 % compared to R4. In addition, the T203A mutation greatly enhances RFK activity and can be used for later modification of FAN/FAD-producing strains. Although there have been studies on enhancing RF production by reducing the enzymatic activity of FADS and on the key site of the RFK active center, the present study is, to the best of our knowledge, the first to target mutations in key amino acid residues of the C-terminal RFK module of *Ec*FADS to enhance RF production.

## Data availability

The supplementary figures are available at the end of this manuscript.

## CRediT authorship contribution statement

**Bing Fu:** conducted the experiments. **Meng Chen:** conducted the experiments. **Xianfeng Bao:** conducted the experiments. **Jiajie Lu:** conducted the experiments. **Zhiwen Zhu:** analyzed the data and drew the corresponding image. All authors read and approved the manuscript. **Fuyao Guan:** analyzed the data and drew the corresponding image. All authors read and approved the manuscript. **Chuyang Yan:** analyzed the data and drew the corresponding image. All authors read and approved the manuscript. **Peize Wang:** analyzed the data and drew the corresponding image. All authors read and approved the manuscript. **Linglin Fu:** analyzed the data and drew the corresponding image. All authors read and approved the manuscript. **Ping Yu:** conceived and designed the experiments, and wrote the manuscript.

## Declaration of competing interest

The authors declare that they have no known competing financial interests or personal relationships that could have appeared to influence the work reported in this paper.
