# Supplementary material for: Site-directed mutagenesis of bifunctional riboflavin kinase/FMN adenylyltransferase via CRISPR/Cas9 to enhance riboflavin production
Source: Synth Syst Biotechnol. 2024 Apr 16;9(3):503–12. doi: 10.1016/j.synbio.2024.04.011 (PMC11047187; doi:10.1016/j.synbio.2024.04.011)
Supplement: Multimedia component 1 [file mmc1.docx]

**Supplementary materials**

(a)

(b)

Figure S1 Schematic map of the construction of expression plasmids. (a) Construction of plasmid pNEW-AZ. (b) Construction of plasmid pET-*ribF*.

(a)


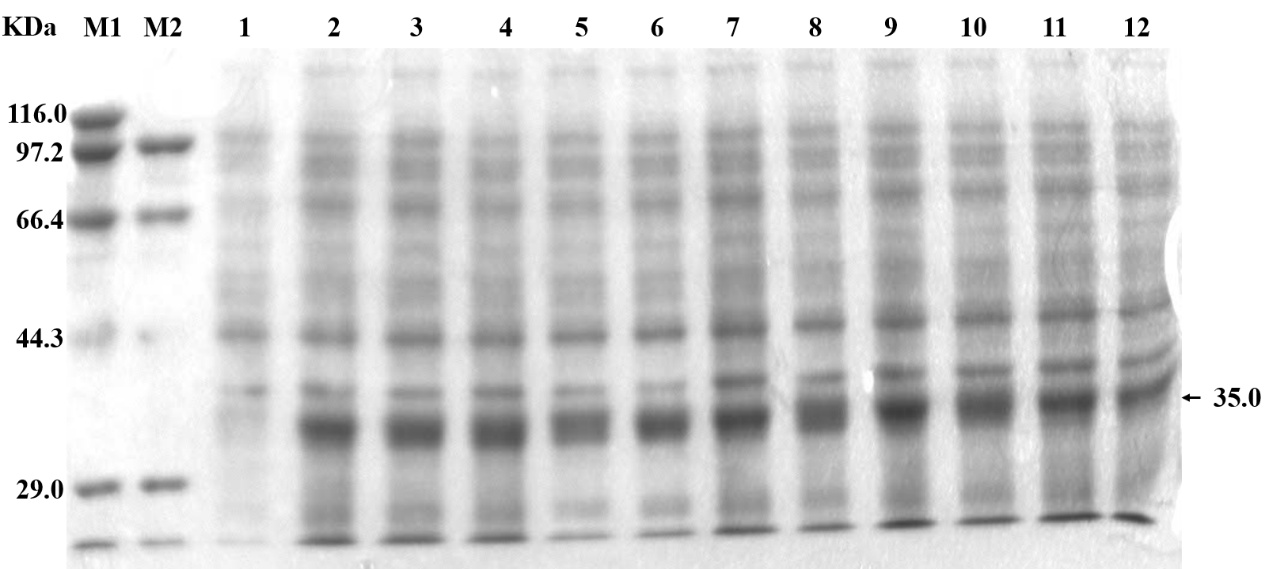


**(b)**


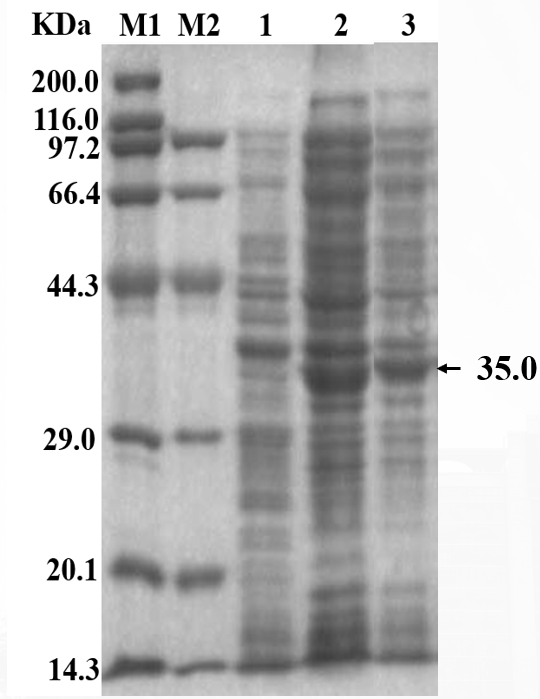


Figure S2 The results of SDS-PAGE of twelve strains’ supernatant

(a) lane M1: protein molecular weight marker (broad); lane M2: protein molecular weight marker (low); lane 1: SDS-PAGE of whole cell protein of *E. coli* BL21(DE3)/pETDuet-1; lane 2-12: SDS-PAGE of supernatant of strains BF, B203A, B203D, B204, B210, B258A, B258D, B259, B260, B261, and B299.

(b) lane M1: protein molecular weight marker (broad); lane M2: protein molecular weight marker (low); lane 1: SDS-PAGE of whole cell protein of *E. coli* BL21(DE3)/pETDuet-1; Lane 2-3: SDS-PAGE of supernatant of strains B303A, and B303D.

(a)


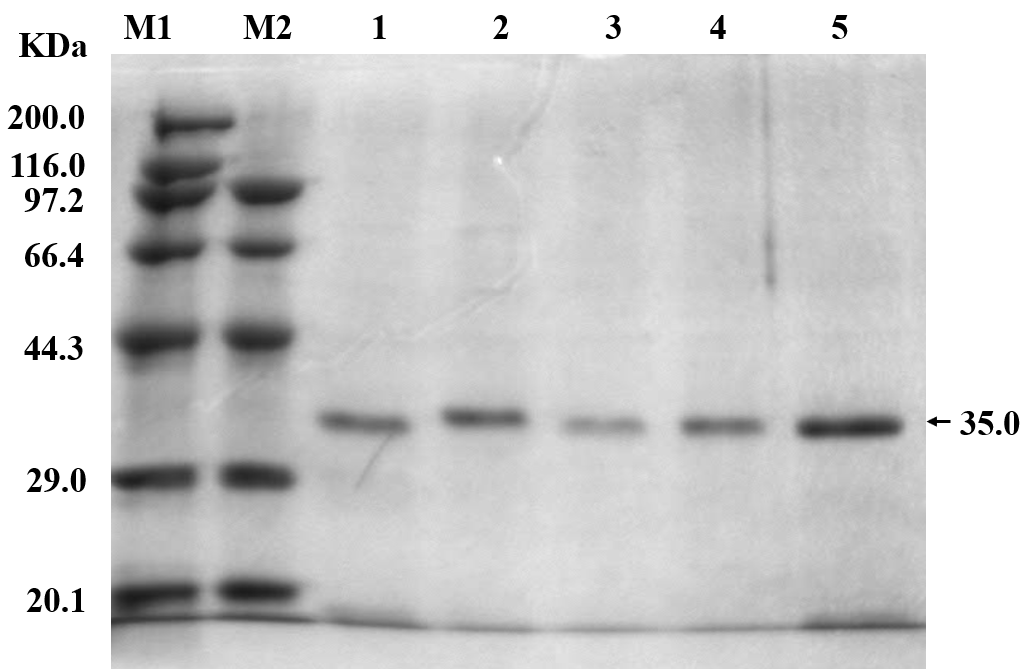


**(b)**


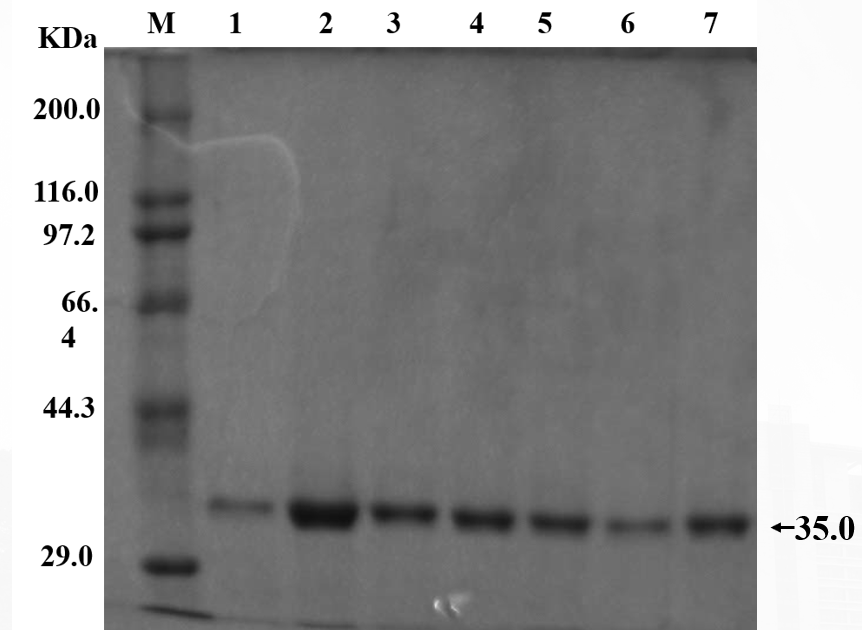


Figure S3 The SDS-PAGE result after Ni^2+^-NTA column purification

(a) lane M1: protein molecular weight marker (broad); lane M2: protein molecular weight marker (low); lane 1-5: SDS-PAGE result of protein from strains BF, B203A, B203D, B204, and B210 after nickel column purification.

(b) lane M: Protein Molecular Weight Marker (Broad); lane 1-7: SDS-PAGE result of protein from strains B258A, B258D, B260, B261, B299, B303A, and B303D after nickel column purification.


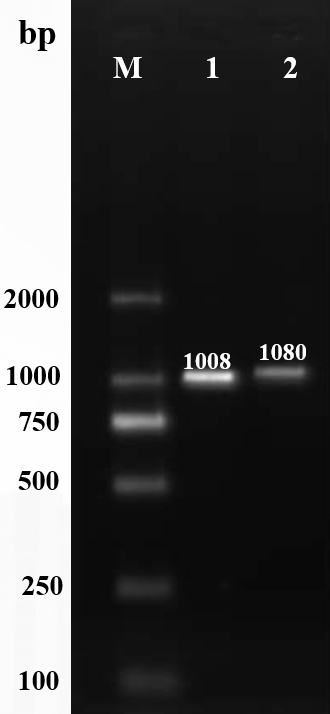


Figure S4 Results of colony PCR validation of the engineered strain with native *ribF* replaced by *ribCopt*.

lane M: DL2000 DNA Marker; lane 1: PCR result for colony of negative strain; lane 2: PCR result for colony of positive strain.
